# The Negative Influence of Air Travel on Health and Performance in the National Basketball Association: A Narrative Review

**DOI:** 10.3390/sports6030089

**Published:** 2018-08-30

**Authors:** Thomas Huyghe, Aaron T. Scanlan, Vincent J. Dalbo, Julio Calleja-González

**Affiliations:** 1 BC Oostende, Oostende 8400, Belgium; 2 Human Exercise and Training Laboratory, School of Health, Medical and Applied Sciences, Central Queensland University, Rockhampton 4702, Australia; a.scanlan@cqu.edu.au (A.T.S.); v.dalbo@cqu.edu.au (V.J.D.); 3 Physical Education and Sport Department, University of Basque Country (UPV-EHU), Vitoria-Gasteiz 48940, Spain; Julio.calleja.gonzalez@gmail.com

**Keywords:** NBA, athletic performance, fatigue, circadian rhythm, injury, sleep

## Abstract

Air travel requirements are a concern for National Basketball Association (NBA) coaches, players, and owners, as sport-based research has demonstrated short-haul flights (≤6 h) increase injury risk and impede performance. However, examination of the impact of air travel on player health and performance specifically in the NBA is scarce. Therefore, we conducted a narrative review of literature examining the influence of air travel on health and performance in team sport athletes with suggestions for future research directions in the NBA. Prominent empirical findings and practical recommendations are highlighted pertaining to sleep, nutrition, recovery, and scheduling strategies to alleviate the negative effects of air travel on health and performance in NBA players.

## 1. National Basketball Association: Schedule and Travel Requirements

The National Basketball Association (NBA) is the premier basketball league in the world [1,2] and in recent years a greater emphasis has been placed on player safety [3,4]. In regard to player safety, there has been increased attention in the areas of training load [3,5] as well as schedule and travel requirements [5]. In an attempt to reduce the training load and schedule requirements of players, the NBA has modified the preseason schedule. Prior to 2017, NBA teams played eight preseason games across 3–4 weeks in preparation for the regular season [6,7]. Since the 2017–2018 season, the NBA season has consisted of four to six preseason games played across 3–4 weeks followed by an 82-game regular season played across 26 weeks (177 days). During the regular season, each team plays two to five games per week (~3.2 games per week) [1] with games lasting an average duration of 2 h and 15 min [2]. NBA teams rarely practice during the season and practices that occur are typically less than 1 h [1,2]. In response to teams resting players during back-to-back (two games within a 2-day span) games [8], the league extended the duration of the regular season by 7 days with the purpose of scheduling fewer back-to-back games [6]. During the 2017–2018 season, NBA teams played an average of 14.4 ± 0.9 back-to-back games, which was the lowest on record compared to any previous season in the NBA [2]. Furthermore, the 2017–2018 NBA season marked the first season in NBA history in which no team played four games in 5 nights [6]. Despite adjustments to the NBA schedule, air travel demands remain high due to the geographical span of teams across four time zones (eastern, central, mountain, and western). In this regard, NBA players spend more time above 30,000 ft than athletes competing in all other team sports in the United States of America (USA) [7]. Air travel requirements are a concern for NBA coaches, players, and owners, as research has demonstrated short-haul flights (≤6 h) increase injury risk [2,9,10,11,12,13] and impede performance [9,14,15,16,17,18,19,20]. Competing in away games has been reported to significantly increase regular season injury risk in a sample of 1443 NBA players between 2012 and 2015 [9]. Specifically, 54% of regular season injuries occurred in players playing games away from home, which was significantly greater than the expected injury rate for away games of 50% (*p* < 0.05) [9]. Furthermore, the direction of air travel should be considered by NBA teams, as traveling westward exacerbates reductions in performance [14,21]. In a sample of 8495 NBA games between 1987 and 1995, west coast teams scored four more points per game (*p* < 0.05) when traveling to the east coast than east coast teams scored when traveling to the west coast [21]. Furthermore, NBA teams traveling eastward had a winning percentage of 45.4% compared with 36.2% for teams traveling westward (*p* < 0.001) between 2010 and 2015 [14]. The increased difficulty of traveling westward across the USA to compete has also been reported in the National Football League and the National Hockey League [14]. Westward travel is likely more difficult since performance tends to peak in the late afternoon and players traveling from west to east tend to play games closer to their circadian peaks given most NBA games are played at night.

## 2. The Impact of Travel Fatigue on Performance

Frequent air travel can negatively affect hydration status, nutritional behaviors, sleep quality, and sleep quantity, thus extending the time for sufficient recovery between games and/or training in athletes [15]. As a result, air travel should be considered as an additional stressor imposed on NBA players in conjunction with competition and training schedules [15], especially when less than 72 h of rest is experienced between games [21,22]. 

One of the main consequences associated with frequent air travel exposure is “travel fatigue”. Travel fatigue refers to feelings of disorientation, light-headedness, gastrointestinal disruption, impatience, lack of energy, and general discomfort that follow traveling across time zones [13]. The magnitude of travel fatigue depends on many factors such as regularity, duration, and conditions of travel [13]. Specific causes of air-related travel fatigue include:

- Prolonged exposure to mild hypoxia [16,23,24].
- Difficulties in standing, walking, and moving around due to limited room inside the air cabin.
- Reduced air quality in the cabin, which may impair immune function [12].
- Dry cabin air and low hypobaric pressure potentially causing dehydration [25].
- Prolonged sitting in a cramped position reducing mobility and flexibility [10,16].
- Disruption of routines (e.g., eating and sleeping) [26].
- Noise of plane and cabin (e.g., sleep disturbance) [16].
- Formalities of air travel may induce negative mood states [26].

A primary issue regarding air travel occurs as a result of significant reductions in oxygen saturation, which has been found to decrease significantly from 97% at ground level to 93% at cruising altitude (*p* < 0.05) [24]. This finding is significant, as oxygen saturation levels of 93% could prompt physicians to administer supplemental oxygen in hospital patients [24] and thus would slow muscle recovery [27]. One study examined the effects of air travel from the east coast to the west coast of the USA on physiological performance measures, sleep quality, and hormonal alterations [28]. However, it is important to note the following: participants used in this investigation were not athletes, a simulated sporting event most closely related to demands experienced during soccer was administered, and there was no non-exercise (control) group. However, air travel induced jet lag symptoms, which resulted in decreased sleep quality and was paired with significantly increased melatonin levels on flight days (travel from east to west coast and travel from west to east coast) [28]. The authors also examined markers of skeletal muscle damage, but since a non-exercise control was not included in the investigation meaningful interpretations of the data cannot be determined [28]. 

When flying across two or more time zones, symptoms of travel fatigue can remain up to 2–3 days after arrival [13]. The physiological and perceptual stressors associated with flying across one or more time zones may alter sleep patterns in athletes [12]. In particular, short-haul air travel has been reported to impair athletic performance due to the development of an inefficient internally-driven circadian rhythm (i.e., sleep deprivation or disorientation between the circadian system and the environment) [29]. In this sense, NBA players may experience difficulty sleeping at night and excessive daytime sleepiness when traveling across multiple time zones. Subsequently, the greater the number of time zones travelled, the more difficult it is for an athlete to adapt to a new time zone. For example, a 2-h time zone shift may cause marginal disruption to the circadian rhythm, but a 3-h time zone shift (e.g., NBA players traveling coast to coast within the USA) can cause a significant desynchronization of circadian rhythm [13]. Therefore, it is recommended that NBA players focus on physical activity, eating, and social contact during daylight in their new time zone in order to resynchronize their circadian rhythm, especially when traveling from coast to coast [13].

The circadian rhythm plays a critical role in sports performance [13,19,30,31]. When an athlete’s circadian rhythm is synchronized with the environment, the athlete should achieve optimal performance during late afternoons and early evenings [19]. Considering air travel can cause an athlete’s circadian rhythm to become unsynchronized with the environment, air travel may contribute to the home court advantage in the NBA [32,33], as the body’s core temperature (an endogenous measure of circadian rhythm) takes approximately 1 day for each time zone crossed to adapt completely to the new time zone [13,34]. Consequently, the number of time zones traveled plays a critical role in the magnitude of travel fatigue [13]. 

The regularity, duration, and direction of air travel, combined with in-cabin conditions, likely predisposes NBA players to travel fatigue [13]. In turn, travel fatigue can have deleterious effects on player recovery and subsequent performance, particularly when scheduled soon after practices or games. Consequently, it is recommended that recovery and practices administered before and after air travel are modified to account for travel fatigue, especially considering the travel direction and flight duration experienced.

## 3. Scheduling and Recovery Opportunities

Besides the direction and duration of air travel, the home court advantage is also influenced by the quantity of rest NBA teams attain prior to games [35]. In particular, a consistent advantage was recorded when a team had more than 1 day of rest between games (the home team’s score increased by 1.1 points per game and the away team’s score increased by 1.6 points per game) in a sample of 8495 regular season NBA games between 1987–1995 [21]. Moreover, average total scores (home and away teams) were highest when 3 days of rest were encountered between games with data collected from the 1987–1995 seasons [21]. Consequently, the negative influence of air travel during an NBA season may be mitigated by incorporating supplemental days to recover from games. 

An optimal recovery window of 72 h following games and practices is needed for an athlete or team to return to optimal levels of performance [22]. Nevertheless, the NBA schedule dictates condensed game schedules that necessitate compressed training schedules, which may inhibit access to active rest days to fully recover from accumulated physical and psychological stress induced by NBA games and practices. Consequently, NBA teams are often obligated to intervene with various ergogenic practices in an attempt to speed up the recovery process, such as whole body cryotherapy, compression tights, cold water immersion, contrast water therapy, and soft tissue massage [36]. While these commonly employed recovery practices, including compression tights [37], cold water immersion [38], and massage [39], have been investigated in various samples of basketball players, no data are available specifically in NBA players. Therefore, more research is needed to ascertain if these recovery practices benefit NBA players across the season.

Another factor to consider in reducing injury risk and optimizing performance in the NBA is the total amount of in-game minutes accrued by each player. While coaches have presumed withdrawing high-minute players from entire games may reduce injury risk and enhance performance, a tactic which is often seen nearing the conclusion of the regular season, data to support this approach is lacking. In fact, existing data revealed the average minutes played per game did not influence on-court performance or injury risk (*p* < 0.001) in 811 NBA players competing between 2000 and 2015 [8,9]. However, it should be noted these data are not reflective of performance and injury risk in players who were rested for entire games but rather are indicative of players completing reduced game minutes. Subsequently, future studies are needed to examine the consequences and confirm the efficacy of resting high-minute players for entire games in the NBA. 

Scientific information about the specific demands of air travel on performance and health in professional team sports is scarce, with research existing in soccer [40] and rugby [41], which may not directly apply to the NBA. Therefore, research is needed to understand the impact of air travel on player health and game performance across the season in the NBA. Future research on the influence of air travel in NBA players should focus on the identification of causes and symptoms of travel fatigue as well as interventions to mitigate the effects of air travel on player health and performance.

## 4. Conclusions and Future Research

The NBA travel schedule induces misalignments in circadian rhythm that cannot be avoided. Air travel across three time zones has been reported to induce susceptibility to travel fatigue [18,29,42,43,44], increase injury risk [13,29,41], and reduce game performance [13,14,17,29,32]. NBA schedule-makers and teams may succeed in mitigating the negative effects of air travel from coast to coast on sleep by implementing up-to-date, evidence-based strategies applied in other professional sports, such as blue light exposure in the morning and red light exposure in the evening, in order to resynchronize the circadian rhythms of players [45]. Other strategies include the ingestion of a high-carbohydrate, low-protein meal in the evening, which may enhance serotonin production to promote drowsiness and sleep [19,46], or the ingestion of a high-protein, low-carbohydrate meal in the morning, which may increase the uptake of tyrosine and its conversion to adrenaline, which elevates arousal and promotes alertness [44,46]. However, future studies are required to evaluate the efficacy of the abovementioned strategies in NBA players.

Despite recent schedule modifications and an increased awareness of the potential negative consequences of air travel on the health and performance of NBA players, there is still a need to implement effective strategies to address issues with sleep and travel fatigue to promote greater equity across western and eastern teams. Future research exploring various aspects of regularity, duration, directions, and conditions of air travel [13] in one or multiple NBA seasons can help identify origins of fatigue in players. Consequently, a holistic approach to future research is recommended, with some potential topics of interest encompassing descriptive and intervention-style studies.

First, it is important to understand the impact of air travel on NBA players at an individual level, given that NBA players often experience time zone transitions, which have been found to increase injury risk [9,41] and hinder performance [15,19,21,40,42,47]. Considering frequent time zone transitions often disrupt the circadian rhythm in athletes [15,16,19,26,42,43], future studies may focus on the measurement of salivary melatonin onset, adrenaline concentrations, and body temperature, as these are critical biomarkers of circadian rhythm [19,48]. Measurement of these biomarkers would provide insight into how each player individually adapts to air travel throughout the NBA season. Consequently, NBA performance support staff may then apply individualized approaches to training and game preparation to combat the negative impact of air travel.

Second, examination of various ergogenic aids will provide a better understanding of practices that may enhance physiological and perceptual responses to air travel in NBA players. For instance, nutrition [49] and hydration [49] are fundamental aspects underpinning circadian rhythm. Therefore, analyzing and comparing the hormonal responses of NBA players adopting different diets may provide NBA coaches and support staff with further insight into beneficial nutritional strategies for coping with air travel in the NBA. 

Third, in order to mitigate the negative impact of air travel on mood state, it is recommended that each player’s psychological and psycho-sociological reactions to air travel should be monitored during the season. For instance, comprehensive psychometric questionnaires such as the Acute Recovery and Stress Scale (ARSS) [50] and the REST-Q Sport [51] have been established as logical, practical, and versatile tools to measure self-perceived travel fatigue in professional team sports [50,51]. Considering the time constraints in the NBA, shorter customized versions of these questionnaires can be completed on a daily basis [52], which have been reported to be valid and reliable in elite Australian Rules Football [53]. However, further research is necessary to provide normative standards, especially with a focus on individual interpretations, recommendations, and compliance in NBA players. 

Finally, considering that skeletal muscle and connective tissues become shortened during flights and may stiffen, it is recommended for players to avoid sitting the entire trip, and instead, walk around the cabin every hour, unless they are asleep or advised not to do so by flight staff [46]. With a tentative agreement between the NBA and Delta Airlines charters, walking inside the air cabin should be attainable, as most NBA teams (27 out of 30 teams) fly with private jets of Delta Airlines (including A319s and Boeing 757-200s) with almost 50 percent more cabin space than standard planes [54]. This cabin space allows most NBA players, who possess an average stature of 6 feet and 7 inches, to have more freedom to stand erect during air travel [54]. Additionally, simple stretching exercises can be applied while in the seat or in the cabin, which could help relax muscles while increasing blood flow and delivering oxygen and other nutrients to muscles [27,46]. As a result, stretching may reduce the negative effects of air travel on flexibility and skeletal muscle recovery. Consequently, future studies are encouraged to examine the efficacy of these in-flight travel strategies in NBA players.
